# Dystopic Os Odontoideum With Chronic Posterior Atlantoaxial Subluxation: A Case Report

**DOI:** 10.7759/cureus.72749

**Published:** 2024-10-31

**Authors:** Mohamed K Elbana, Asmaa E Elgebally

**Affiliations:** 1 Maxillofacial Surgery, Aberdeen Royal Infirmary, Aberdeen, GBR; 2 Diagnostic and Interventional Radiology Department, Faculty of Medicine, Medical Research Institute, University of Alexandria, Alexandria, EGY

**Keywords:** atlantoaxial subluxation, cervical mri, myelopathy, occipitocervical, os odontoideum

## Abstract

Os odontoideum is considered a rare cervical spine anomaly. Depending on its type, it may or may not result in atlantoaxial instability, which can lead to anterior subluxation (the most common type) or posterior subluxation, with the latter being extremely rare. Posterior atlantoaxial subluxation is particularly dangerous, as it can progressively lead to myelopathy through various mechanisms and may ultimately result in quadriplegia or even death.

## Introduction

Os odontoideum (OO) is an uncommon anatomical variation found in the upper cervical spine, initially documented by Giacomini in 1886. This anomaly is identifiable through radiological imaging as an oval or round-shaped bone fragment with smooth, circular cortical edges. It represents an underdeveloped odontoid process (dens) that does not connect to the body of the second cervical vertebra (C2). Typically, the bone fragment migrates upward to the anticipated location of the odontoid tip. There are two main anatomical types in which this anomaly can manifest: orthotopic, where the bone fragment is situated in the usual odontoid position, and dystopic, where it is positioned near the occiput in the region of the foramen magnum [1]. Radiological studies have shown that OO is typically about half the size of a normal odontoid process. However, there are unusually small and cephalic ossicles in some instances, making diagnosis challenging with standard imaging methods such as X-rays or CT scans [2]. OO is often located behind and above the front arch of the first cervical vertebra (C1) and is frequently connected to the front arch by the transverse ligament. One of the main risks associated with OO is forward displacement of the first and second cervical vertebrae, while backward displacement is uncommon. This instability in the joint between the first and second cervical vertebrae can result in a narrowing of the spinal canal in the neck, leading to myelopathy due to compromised blood flow, pressure from bone, or stretching of the spinal cord. Despite its rarity, it has been linked to serious neurological consequences; there have been cases where patients have suffered paralysis after a minor injury, particularly when there is inadequate ligament support for the dens [3].

The complexities of this condition and the challenges in diagnosis and management highlight the importance of thorough evaluation and monitoring of affected individuals. Ongoing research and awareness are crucial for better understanding its causes, prevalence, and best treatment strategies, especially since many cases go undetected until symptoms appear. A multidisciplinary approach is essential for effectively managing patients to prevent serious complications and improve clinical outcomes.

## Case presentation

We report an unusual case of a 42-year-old Egyptian female who was diagnosed clinically and radiologically with the dystopic type, resulting in posterior atlantoaxial subluxation and progressive myelopathy.

She has had symptoms of headache and difficulty in walking for the past four years. Her history began with subtle, persistent symptoms, including headache, limited neck mobility, and difficulty walking since childhood, progressing over time. The symptoms became disabling over the last four years. She was initially diagnosed with a functional neurological disorder and was treated with analgesics. As the clinical signs did not improve, she was subsequently referred to neurosurgery, complaining of headache, neck pain, limited neck mobility, weakness, and numbness in the upper and lower limbs, more noticeable on the right side, with no history of any previous detectable trauma.

Physical examination revealed very limited neck mobility, with maximal rotational neck movement reaching only 15° on both the right and left sides. The patient exhibited axial neck pain, spastic gait, and increased muscle tone in all limbs, more pronounced on the right side. Manual muscle testing revealed grade 3 muscle power in all limbs. The extensors in the upper limbs were weaker than the flexors, while the flexors in the lower limbs were weaker than the extensors. Generalized hyperreflexia was observed in both the upper and lower limbs, which was more prominent on the right side. Sensory examination showed hypoesthesia in the upper and lower limbs, again more marked on the right side. Babinski and Hoffman’s reflexes were positive bilaterally, with a stronger response on the right side. Additionally, the patient reported intermittent urinary retention and constipation.

**Figure 1:**
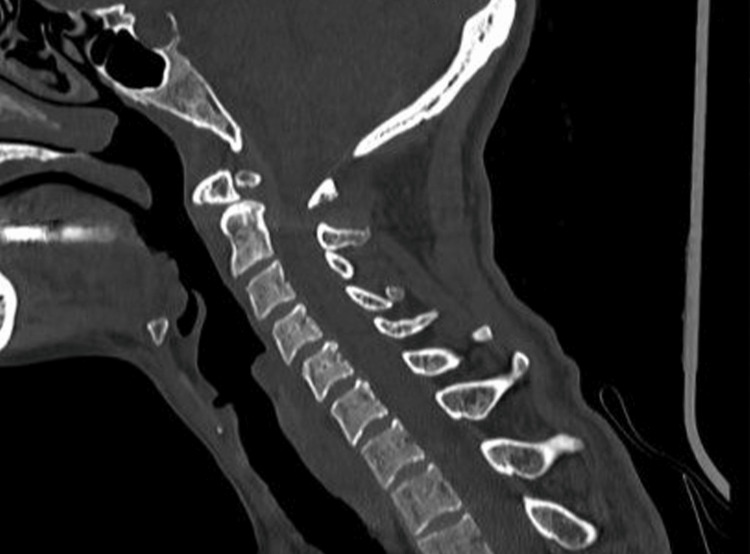
Computed tomography (CT) demonstrated straightening of the examined cervical spine, reflecting paravertebral muscle spasm.

**Figure 2:**
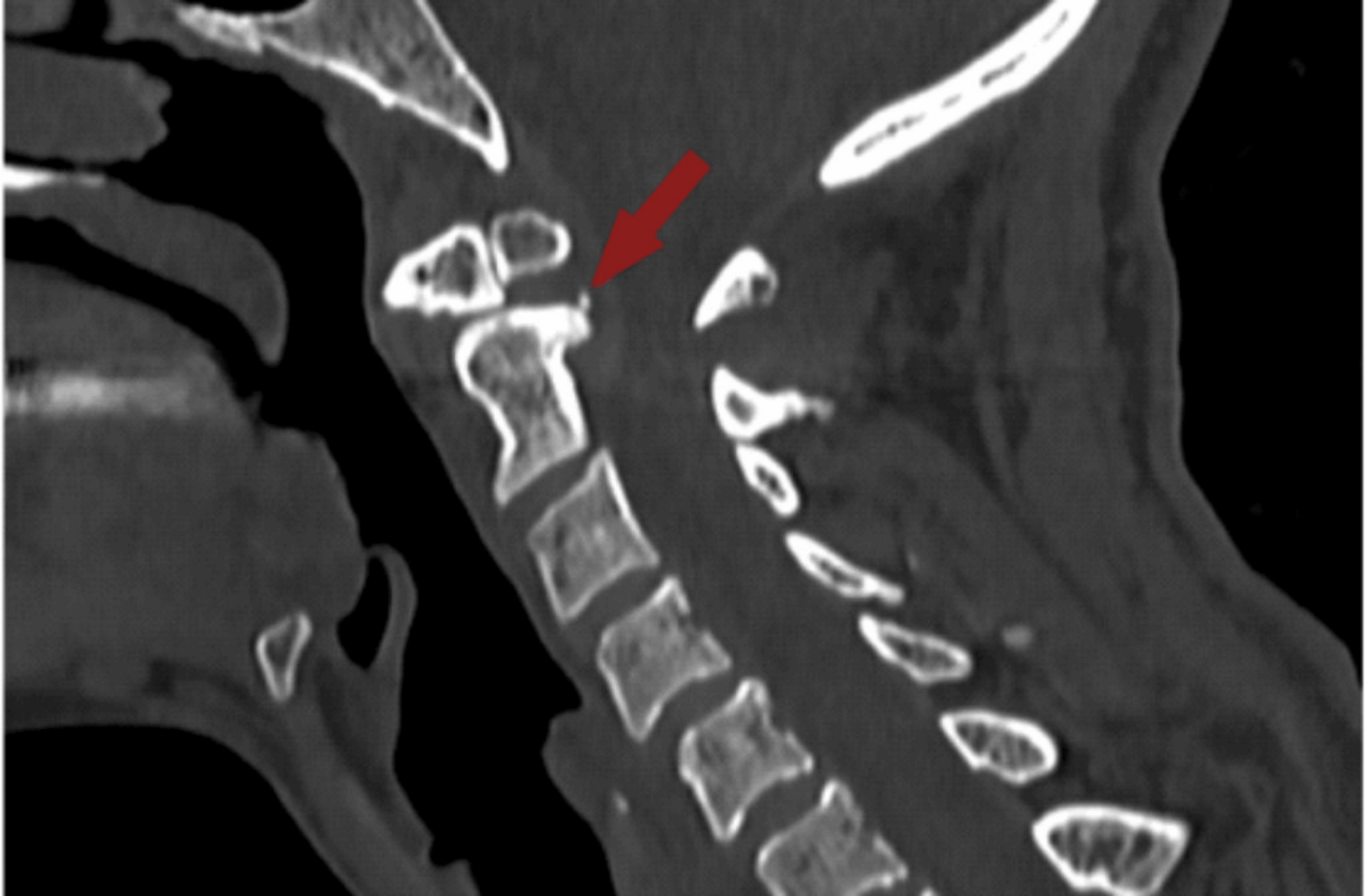
CV2 shows the dystopic type of os odontoideum and posterior atlantoaxial subluxation, with a few osteophytes indicated by the red arrow.

**Figure 3:**
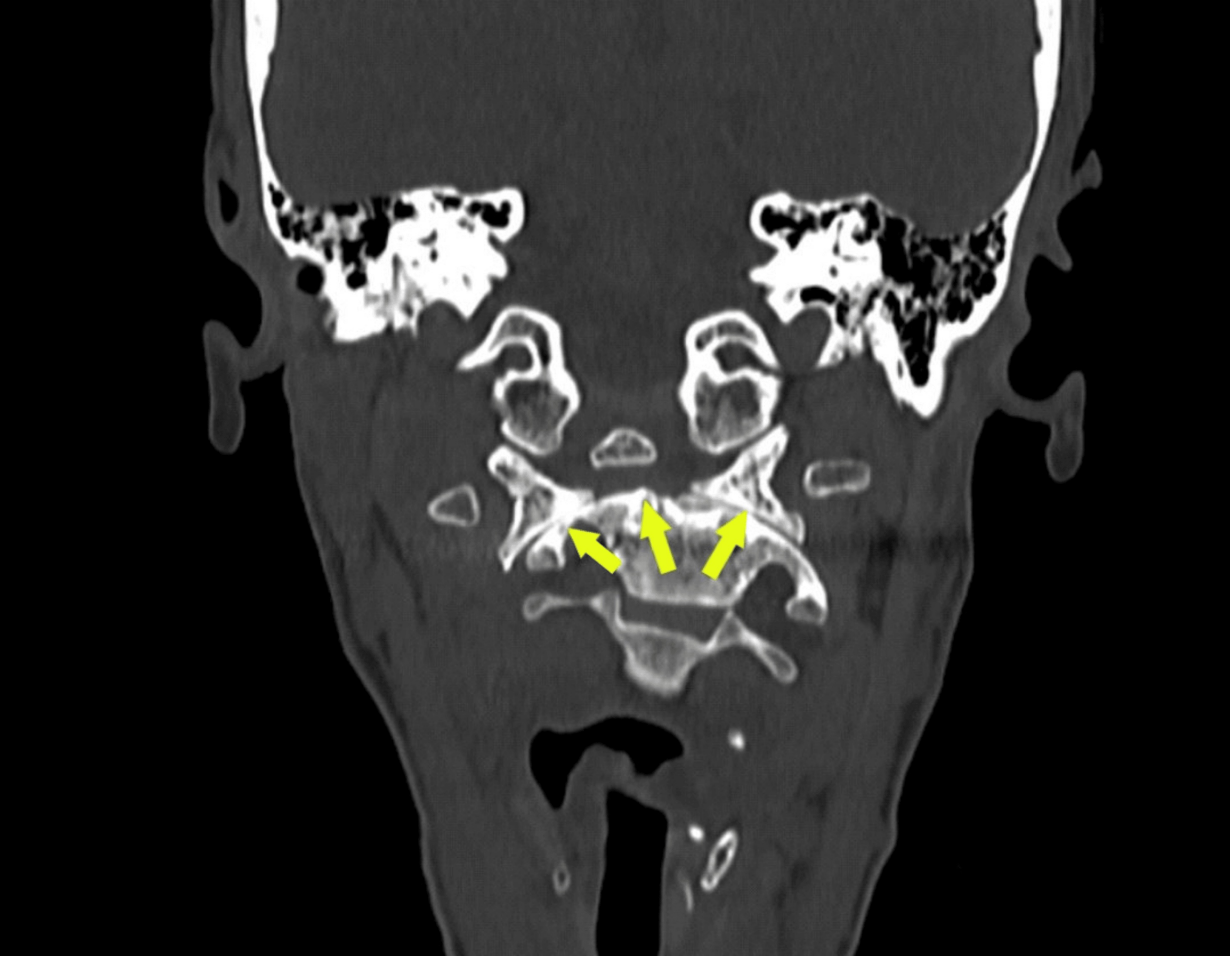
Lateral atlantoaxial subluxation is noted on the right side.

**Figure 4:**
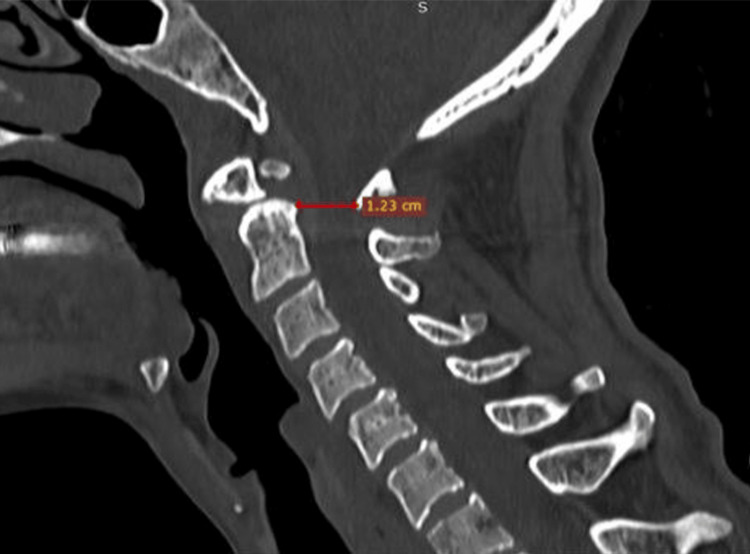
The measurement of the posterior atlantodens interval (PADI) is 12 mm.

**Figure 5:**
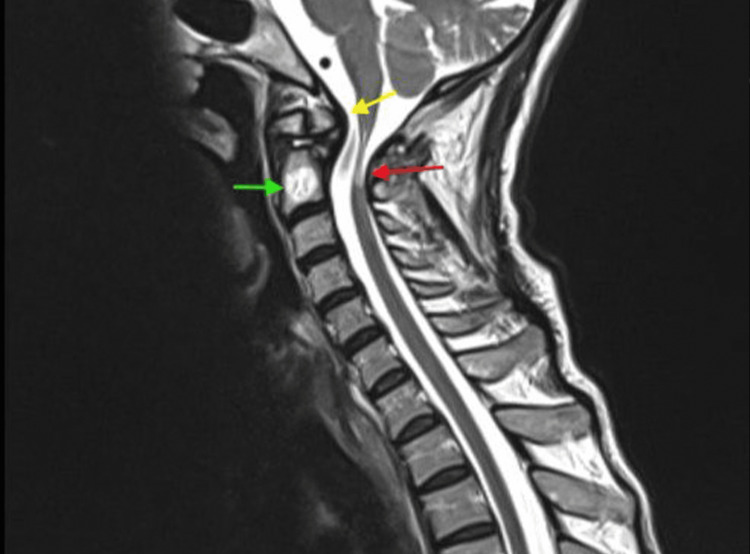
Magnetic resonance imaging (MRI) The MRI scan shows a thickened tectorial membrane (yellow arrow), marked focal atrophy of the most proximal portion of the cervical cord at the CV1 and CV2 levels, displaying an intramedullary T2 hyperintense signal (red arrow), significant spinal cord compression at the same level, and a vertebral body hemangioma noted at the CV2 vertebral body (green arrow).

A CT scan and MRI were performed promptly due to the patient’s critical presentation (Figures 1-5).

Laboratory findings, including serum, urine, and serological tests for rheumatoid factor, were within normal limits.

The patient was managed conservatively with a neck collar until preparation for surgical intervention; however, she refused surgical treatment despite medical advice regarding the risks of not proceeding with surgery.

## Discussion

**Figure 6:**
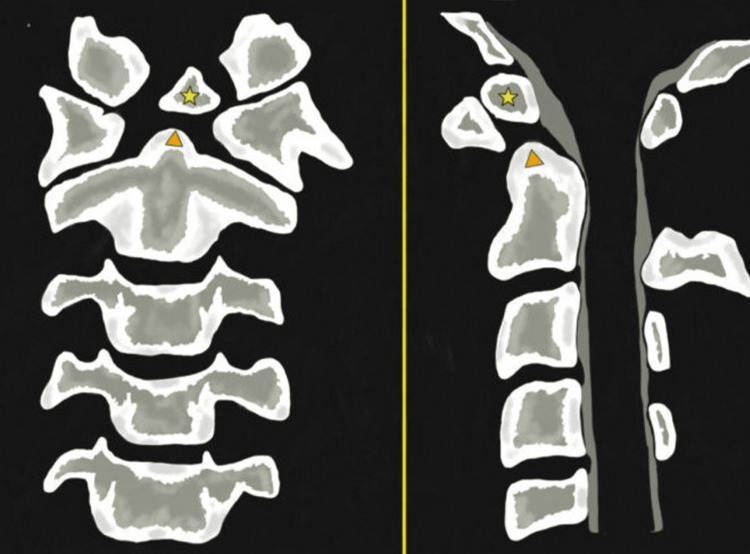
Os odontoideum. Anterior-posterior (left-sided image) and lateral (Right-sided image) views. They show an oval or round-shaped ossicle with smooth circumferential cortical margins (yellow star) representing a hypoplastic odontoid process (Orange triangle) that has no continuity with the C2 vertebral body. It is often attached to the anterior arch of C1 through an intact transverse ligament.

Progressive myelopathy secondary to posterior atlantoaxial instability caused by the dystopic type is a rare condition that necessitates accurate diagnosis due to its serious clinical presentation, which can range from occipitocervical pain to death (Figure 6).

A shortage of literature exacerbates the controversies surrounding the etiology, natural history, and management of [4]. The presentation of both categories varies, spanning from incidental discoveries without symptoms to neurological impairment [5]. These symptoms are caused by instability between the C1 and C2 vertebrae. This instability can also result in vertebrobasilar ischemia, occipital-cervical pain, and atlantoaxial dislocation (AAD) [6,7]. Patients who have compromised joint stability at the beginning of the disease may experience neural and vascular compression symptoms, which puts them at risk for sensory disorders, cervical myelopathy, quadriplegia, other motor disturbances, cerebellar infarction, or brainstem damage [8,9]. In extreme circumstances, it may cause the respiratory center to become paralyzed and lead to abrupt death [10].

Our case presented with neurological symptoms indicative of severe myelopathy, which necessitated adequate radiological examination to reach an accurate diagnosis. She was referred for cervical CT assessment and MRI of the cervical spine. After the diagnosis of posterior atlantoaxial subluxation and the resultant severe myelopathy, we retrospectively performed dynamic flexion-extension plain radiographs to assess the stability of the cervical spine.

Due to the lack of extensive epidemiological studies and the incidence of asymptomatic development in individuals, the precise prevalence of is unknown [11].

Regarding the etiology of OO, there are two primary theories: congenital and traumatic. Each has its own supporting and limiting points. OO presents a wide spectrum of clinical manifestations, with many cases discovered incidentally (Table 1) [4].

**Table 1:** Presentation and Diagnostic Imaging of Os Odontoideum AAI: atlantoaxial dislocation; CT: computed tomography; MRI: magnetic resonance imaging

OO type	Presentation	Imaging modality
Asymptomatic	Absence of compression/Instability or radiographic evidence of atlantoaxial instability (AAI)	Plain radiographs + CT or MRI for confirmation of prophylactic surgery
Symptomatic	Occipitocervical pain, restricted neck movements, numbness, weakness, gait impairment, myelopathy, paresis, tetraplegia, central cord syndrome, bulbar signs, intracranial symptoms, brainstem damage, cerebellar infarction, cervical vertigo, seizures, and vertebrobasilar ischemia.	Plain radiographs followed by CT and MRI, along with multiplanar reconstruction and angiography for surgical planning, as well as kinetic MRI for assessing spinal cord signal changes.

Even though the clinical presentation of (OO) can mimic many other disorders, it is important to differentiate the condition accurately in order to consider appropriate treatment options [12].

The differential diagnosis includes acute fracture of the odontoid process, where a clear history of trauma, along with multiple adjacent fractures and soft-tissue injury, will help establish an accurate diagnosis [4, 5]. Another differential diagnosis would be persistent ossiculum terminale, which occurs when the apex at the secondary ossification center does not unite. The ossicle in this case is smaller than that in OO, is rarely associated with C1/C2 instability, and typically does not require surgical treatment [5,12]. Additionally, degenerative disc disease of the cervical spine should be excluded. This is characterized by generalized spondylodegenerative changes.

Diagnosis of OO can be made using various radiological modalities, which we will discuss from the simplest to the most advanced. X-ray in different views, including the open mouth, anteroposterior (AP), lateral, and dynamic views, allows us to assess the condition, associated subluxation, and atlantoaxial stability [9]. An atlanto-dental interval (ADI) greater than 3-5 mm on flexion-extension films is generally accepted as an indicator of atlantoaxial instability [12]. CT helps define the osseous architecture, with multiplanar reconstruction scans facilitating surgical planning and enabling accurate identification of any associated anomalies [6,12]. The posterior atlantodens interval (PADI), the distance from the posterior surface of the dens to the anterior surface of the posterior arch of C1, is a predictor of paralysis onset if it measures shorter than 14 mm. MRI offers superior soft-tissue resolution and provides more preoperative details on disease and spinal cord compression [5,6,12]. Additionally, due to its high sensitivity, MRI can diagnose common pathologies such as synovial cysts, cervical spondylotic myelopathy, disc prolapses, and spinal cord tumors [13].

Management of OO varies according to the presenting symptoms and whether it is complicated by radiologically proven atlantoaxial subluxation. In asymptomatic cases incidentally diagnosed through radiological procedures for other reasons, conservative management is typically the rule, with prophylactic surgical options discussed with the patient. Conservative management includes clinical and radiological follow-up, and sometimes immobilization using a neck collar may be necessary [5,14]. Several studies have shown that patients with stable OO can benefit from long-term conservative care [5,14]. However, there have also been reported cases of neurological impairment and sudden death [15,16]. As stable cases may deteriorate, aggressive conservative management is advised, along with patient education about potential hazards and recommendations to avoid contact sports [17]. Conversely, patients with radiographic signs of atlantoaxial instability (AAI), dynamic myelopathy, or neurological dysfunction require surgical decompression and stabilization [4,10]. A variety of surgical methods, including anterior and posterior approaches or a combination of both, can be tailored to each unique case [18]. The choice of surgery depends on the anatomical variety of OO in patients, which includes factors such as the location of spinal cord compression, bone quality, and the site of arthrodesis, in addition to other craniovertebral junction morphological characteristics [17]. Transoral decompression may be used in conjunction with any surgical technique when there is irreducible atlantoaxial subluxation or ventral compression [18].

## Conclusions

This case report highlights the rarity and critical nature of dystopic OO with posterior atlantoaxial subluxation, presenting with progressive myelopathy. Accurate diagnosis through radiological assessment is vital, as this condition can be easily misdiagnosed due to its overlapping symptoms with other cervical spine disorders. Misdiagnosis can lead to a delay in appropriate treatment, increasing the risk of severe neurological deterioration, including quadriplegia or even death. Despite the patient’s refusal of surgical intervention, which is generally the recommended course of action in such cases, the importance of early detection and proper management cannot be overstated. Conservative measures may be suitable in stable cases, but surgical stabilization is crucial when neurological impairment and atlantoaxial instability are present. This case underscores the need for heightened awareness of this condition among clinicians to prevent delayed diagnosis and treatment, which could have catastrophic outcomes.
